# The Transmission Dynamics of Tuberculosis in a Recently Developed Chinese City

**DOI:** 10.1371/journal.pone.0010468

**Published:** 2010-05-03

**Authors:** Peng Wu, Eric H. Y. Lau, Benjamin J. Cowling, Chi-Chiu Leung, Cheuk-Ming Tam, Gabriel M. Leung

**Affiliations:** 1 Department of Community Medicine and School of Public Health, Li Ka Shing Faculty of Medicine, The University of Hong Kong, Hong Kong SAR, China; 2 Tuberculosis and Chest Service, Department of Health, Government of the Hong Kong SAR, Hong Kong SAR, China; Institut Pasteur, France

## Abstract

**Background:**

Hong Kong is an affluent subtropical city with a well-developed healthcare infrastructure but an intermediate TB burden. Declines in notification rates through the 1960s and 1970s have slowed since the 1980s to the current level of around 82 cases per 100 000 population. We studied the transmission dynamics of TB in Hong Kong to explore the factors underlying recent trends in incidence.

**Methodology/Principal Findings:**

We fitted an age-structured compartmental model to TB notifications in Hong Kong between 1968 and 2008. We used the model to quantify the proportion of annual cases due to recent transmission versus endogenous reactivation of latent infection, and to project trends in incidence rates to 2018. The proportion of annual TB notifications attributed to endogenous reactivation increased from 46% to 70% between 1968 and 2008. Age-standardized notification rates were projected to decline to approximately 56 per 100 000 in 2018.

**Conclusions/Significance:**

Continued intermediate incidence of TB in Hong Kong is driven primarily by endogenous reactivation of latent infections. Public health interventions which focus on reducing transmission may not lead to substantial reductions in disease burden associated with endogenous reactivation of latent infections in the short- to medium-term. While reductions in transmission with socio-economic development and public health interventions will lead to declines in TB incidence in these regions, a high prevalence of latent infections may hinder substantial declines in burden in the longer term. These findings may therefore have important implications for the burden of TB in developing regions with higher levels of transmission currently.

## Introduction

While tuberculosis (TB) remains a leading cause of death in many developing countries [1], some developed countries with low incidence and prevalence have begun to investigate measures to eliminate TB [2]–[4]. Hong Kong is an affluent subtropical city with a well-developed healthcare infrastructure but a relatively high TB burden with around 82 cases per 100 000 population in 2008 [5]. Medical developments, demographic changes and socio-economic improvements led to dramatic declines in local age-standardized TB notifications and mortality rates by 56% and 84% between 1960 and 1980, however the decline in notifications has slowed since the late 1980s [5], [6]. We constructed a mathematical model to study the transmission dynamics of TB in Hong Kong between 1968 and 2008 and to explore the potential factors underlying the attenuating declines in incidence. Transmission in our model varies dynamically in proportion to the number of infectious cases, and we estimate key uncertain parameters including the transmission coefficients. By specifically accounting in our model for the routes by which active TB disease developed, we differentiate notifications due to recent exogenous transmission versus endogenous reactivation, with implications for control measures and trends in disease burden in the short- to medium-term future.

## Materials and Methods

### Sources of Data

Tuberculosis has been a notifiable infectious disease in Hong Kong since 1939. Annual age- and sex-specific TB notifications from 1968 to 2008 were obtained from the Department of Health of the Hong Kong government. The TB notification system was re-organized in 1967 [5], leading to a temporary artefactual fluctuation in notification rates in the late 1960s. Annual age- and sex-specific mid-year populations, annual birth rates, and age-, time- and sex-specific death rates from 1961 to 2007 were obtained from official statistics published by the Census and Statistics Department (CSD) of the Hong Kong government. We also adopted the population projections from 2008 to 2018 compiled by CSD [7]–[10]. Data on local recovery rates of newly diagnosed TB cases were reported by the TB and Chest Service of the Hong Kong government [11]. For consistency with local statistics, age-specific incidence rates were standardized to the World Standard Population [12].

### Model Description

**Figure 1:**
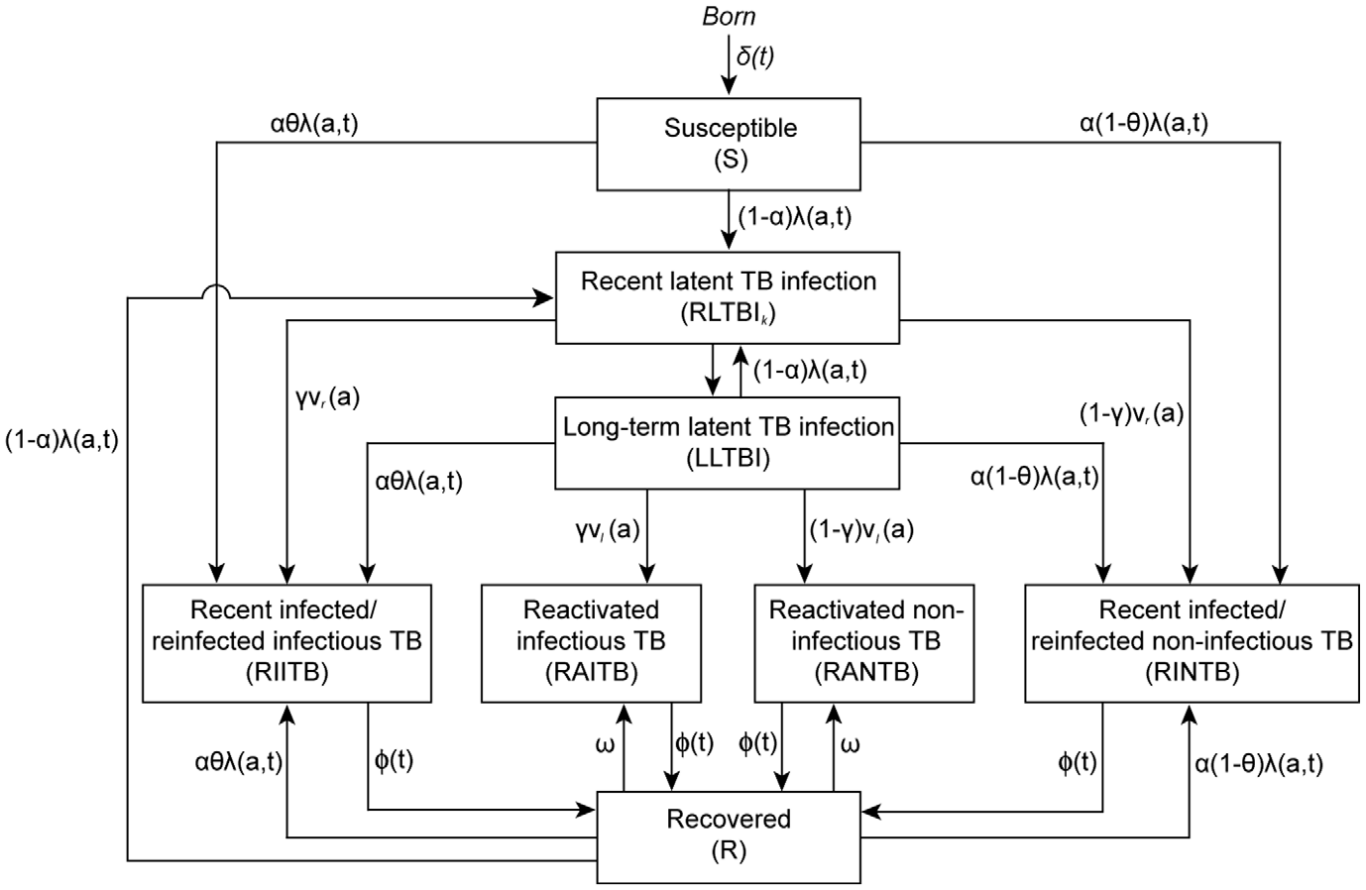
The schematic framework of the TB transmission dynamic model. The absorbing death state is not shown.

We specified a compartmental model for TB [13] and developed the model to explicitly incorporate age structure. The model is composed of eight compartments or states (Figure 1). Individuals are born without infection and are assumed susceptible (S) to infection. An individual who is infected with TB may develop active TB disease (infectious or non-infectious) immediately or may have latent TB infection without presenting any clinical symptoms or signs of active disease. In the latter case, an individual is described by the model as having recent latent TB infection (RLTBI). RLTBI is a transient state and after 5 years of infection the individual in RLTBI transits to the long-term latent TB infection (LLTBI) state. This structure allows individuals in the RLTBI state to have a different rate of progression to active TB disease from those in the LLTBI state.

The route by which active TB disease develops can be classified into one of the two categories, namely recent exogenous infected/reinfected and endogenous reactivated TB disease. Active TB disease which develops within 5 years of first infection or an infection that was not the first ever infection (i.e. a reinfection) is classified as recent infected/reinfected infectious TB (RIITB) or noninfectious TB (RINTB) disease. Active TB disease which develops from an infection which occurred at least five years earlier is classed as reactivated infectious TB (RAITB) or noninfectious TB (RANTB) disease [14]. The progression rate to active TB disease varies for individuals being infected for no more than 5 years or more than 5 years [15], [16]. The separate pathways through RLTBI and LLTBI to, RIITB, RAITB, RINTB or RANTB allow us to quantify the number of active TB cases due to endogenous reactivation and exogenous recent transmission. The force of infection is assumed to be frequency-dependent in the model (Text S1), which is a typical assumption in studying disease transmission dynamics with heterogeneous contacts [17], [18].

Individuals who have had active TB disease, have completed a full course of treatment and have remained free from relapse for at least 24 months are classified as recovered (R). Individuals in the recovered state do not have protective immunity and may later develop active TB disease either via endogenous reactivation or by exogenous reinfection with TB. Individuals in every state may die, represented in the model as an absorbing state - death, with a risk that varies with age and time.

### Model Assumptions

1. TB transmission varies within and between individuals of different age groups to reflect the age structure in the mixing pattern. In our model we classify individuals into three age groups: children (aged from 0 to 15 years), younger adults (aged from 16 to 30 years), and older adults (aged 31 years or older). We used this classification since individuals aged 16–30 years old may be the most active group with more social contacts with each other than any other age groups.
2. The disease progression rates differs for infected individuals in different age groups (Text S1 and Figure S1)
3. Individuals who recover from active TB disease do not have protective immunity against subsequent reinfection [19].
4. Individuals with active TB disease (infectious or non-infectious) cannot be re-infected before recovering from the current disease episode.
5. Individuals in the RLTBI state (having been infected not more than 5 years without an active disease episode) cannot be re-infected before recovering from the current infection, while individuals in the LLTBI state (having been infected more than 5 years without an active disease episode) have the same risk of reinfection as susceptible individuals.

### Model Parameterization, Estimation and Validation

We constructed a demographically appropriate model of the Hong Kong population from 1961 through 2008 by incorporating local data on annual birth rates and death rates, as well as on age- and time-specific changes in the population via immigration and emigration (Text S1). Table 1 defines the fixed parameters of our transmission model, listing the values and ranges that are based on local data where available and otherwise based on data from the literature. Further details of the variables and notations used in the model are provided in Text S1 and Table S1.

**Table 1:** Summary of fixed parameter values used in the model.

Parameter	Units	Parameter value	Range of sensitivity analysis	Source
Period-specific birth rate (*δ(t)*)	/year			[10]
All-cause death rate (*μ(a,t)*)	/person/year			[8]
Recovery rate for infectious or non-infectious TB patients in 1961 (*φ (0)*)	/person/year	0.2	0.1–0.5	[11]
Proportion of developing infectious TB directly from the susceptible, LLTBI or recovered state (*θ*)		0.7	0.50–0.85	[8], [13]
Proportion of developing infectious TB from latent TB infection (*γ*)		0.85	0.50–1.00	
Probability of relapse for recovered patients (*ω*)	/person/year	0.017	0.016–0.018	[11]
The prevalence of latent TB in 1961 (*P_L0_*)		0.7	0.1–0.9	
Ratio of TB prevalence to incidence cases in 1961 (*π_T0_*)		2.5	0.1–5.0	
Relative risk of disease progression in different age groups (*κ(a)*)		See Appendix Figure 2		[15], [16]

In addition to the fixed parameters, we estimated six key parameters for which the values could not be derived from historical data or the literature and were likely to be specific to the local situation (Table 2), namely the infection parameter (*b*), the proportion of new infections directly developing into infectious or non-infectious TB disease (*α*), disease progression rate within the first 5 years after infection (*p_r_*) and after being infected for more than 5 years (*p_l_*) for the reference group (aged 24), and two relative transmission factors in the transmission matrix represented by *M_1_* and *M_2_*, indicating the relative transmission risk within children and within younger adults, respectively, compared to the transmission within older adults (Text S1). We initialized the model in 1961, assumed the prevalence of TB infection in different age groups following a logistic distribution (Text S1 and Figure S2), and used a likelihood function based on a negative binomial distribution to fit the model to age-specific annual TB notification rates from 1968 through 2003. The years from 1961 through 1967 were included as a burn-in period to minimize the influence of the initial state. We used maximum likelihood to estimate the six parameters in the model and evaluated the information matrix numerically to estimate marginal 95% confidence intervals. The model was validated by comparing TB incidence predicted by the fitted model with observed age-specific TB notifications from 2004 through 2008 and by the consistent correlation with the TB transmission dynamics showed in the correlation matrix of the estimated parameters (Table S2). We used the model to project future age-specific TB notifications from 2009 through 2018, with 95% prediction intervals.

**Table 2:** Summary of parameters estimated in the model.

Parameter	Unit	Estimated value	95% confidence interval (CI)
Infection parameter (*b*)	/person/year	14.7	(14.1, 15.7)
Relative transmission factor (*M_2_*)		7.1	(6.4, 7.9)
Relative transmission factor (*M_1_*)		0.19	(0.17, 0.21)
Proportion of new infections directly developing infectious or non-infectious TB from susceptible, LLTBI or recovered state (*α*)		0.013	(0.011, 0.015)
Disease progress rate within the first 5 years after infection for the reference group (aged 24) (*p_r_*)	/person/year	0.0018	(0.0015, 0.0022)
Disease progress rate after being infected for more than 5 years for the reference group (aged 24) (*p_l_*)	/person/year	0.000057	(0.000016, 0.000095)

### Sensitivity Analyses

We performed one-way sensitivity analyses to examine the influence of each of the fixed parameters on the trends in TB notifications predicted by the model. We varied the proportion of active TB disease which is infectious (*θ*), the proportion of active TB disease from latent TB infection (RLTBI or LLTBI) which is infectious (*γ*), the probability of relapse for recovered patients (*ω*), the recovery rate for TB patients in 1961 (*φ(0)*), the prevalence of latent TB in 1961 (*P_L0_*), and the ratio of TB prevalence to incidence cases in 1961 (*π_T0_*). Each parameter was varied between minimum and maximum plausible values as determined from local data or the literature (Table 1).

We performed a multivariate sensitivity analysis based on Latin hypercube sampling to examine the influence of all the fixed parameters in combination [20]. We divided the plausible ranges for each parameter (Table 1) into 100 equiprobable intervals. We then simulated 100 sets of samples in which each variable was drawn randomly and without replacement from these intervals. Each of the 100 sets of fixed parameters was used in place of the original values in the model to explore uncertainties in the total number of notifications, and the proportion due to recent transmission or endogenous reactivation. We also tested the plausibility of alternative relative transmission risk matrix across age groups.

## Results

**Figure 2:**
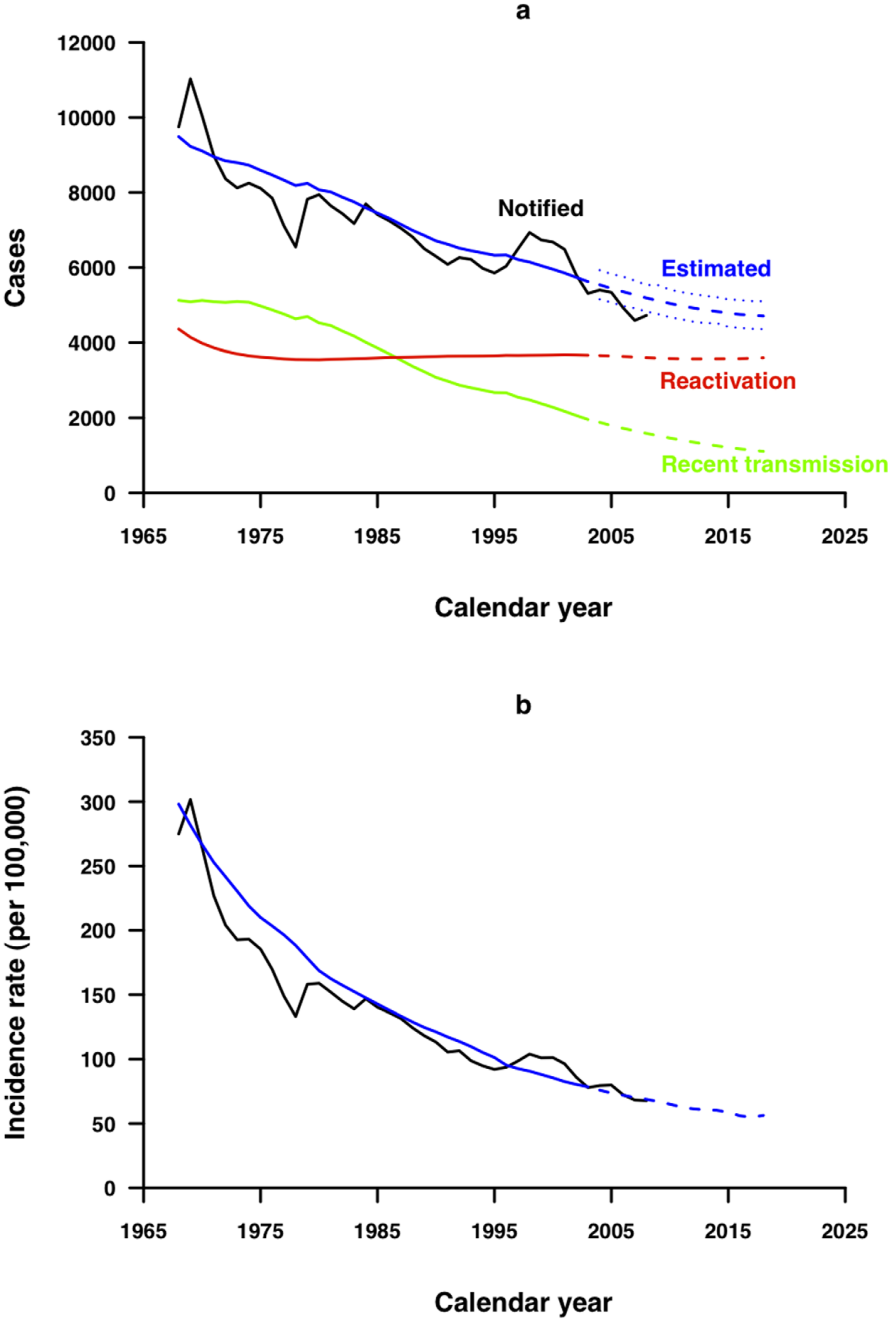
TB incidence in Hong Kong. (a) Observed TB notifications from 1968 through 2008 (black) compared to fitted total (blue), recently transmitted (green) and reactivated (red) TB cases from 1968 through 2003, and predicted total (blue dashed), recently transmitted (green dashed) and reactivated (red dashed) TB cases from 2004 through 2018, with 95% prediction interval (blue dotted). (b) Observed age-standardized TB notification rates from 1968 through 2008 compared to fitted (blue) and age-standardized TB notification rates from 1968 through 2003, and predicted (blue dashed) TB notification rates from 2004 through 2018.

Table 2 lists the parameter estimates and 95% confidence intervals for the six estimated parameters in the model fitted to notifications from 1968 through 2003. The annual number of newly developed active TB cases predicted by the best-fitting model closely matched the trends in total TB notifications as well as the age-standardized notification rates in Hong Kong from 1968 through 2008 (Figure 2). After the 1970s the number of cases due to recent transmission declined much more rapidly than the number of cases due to endogenous reactivation. Between 1968 and 2008 the proportion of annual TB notifications attributed to endogenous reactivation increased from 46% to 70% (Figure 2a). Under the fitted model, notification rates are projected to continue to decline slowly after 2008, with age-standardized incidence rates approaching 56 per 100 000 in 2018.

**Figure 3:**
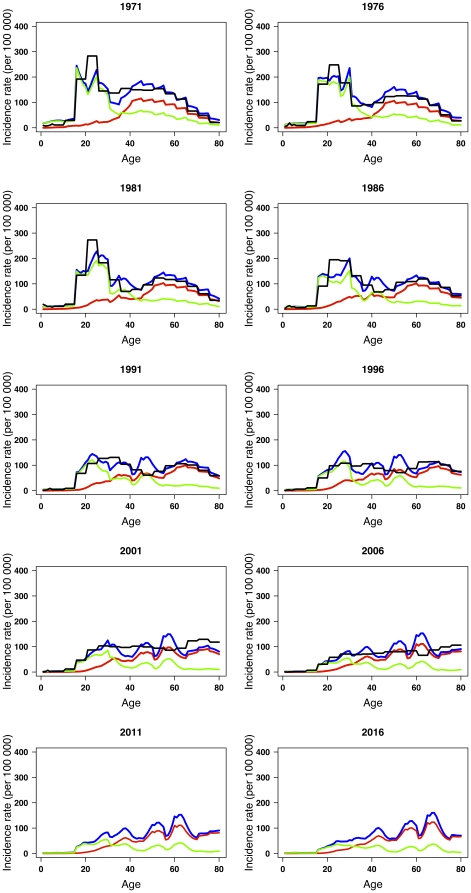
Observed TB age-specific notifications compared to fitted and predicted total (blue), recently transmitted (green) and reactivated (red) TB cases at 5 year intervals from 1968 through 2008, and model predictions for 2011 and 2016.

The model was able to closely match age-specific TB incidence to trends in local notifications from 1968 through 2008 (Figure 3). Throughout the period, most notifications in the older age groups were estimated to be a result of endogenous reactivation, while most cases among teenagers and younger adults were estimated to be attributable to recent transmission. Allowing for age dependency in the risk of disease progression, the estimated lifetime risk of TB disease for infected individuals was around 1%–2%.

**Figure 4:**
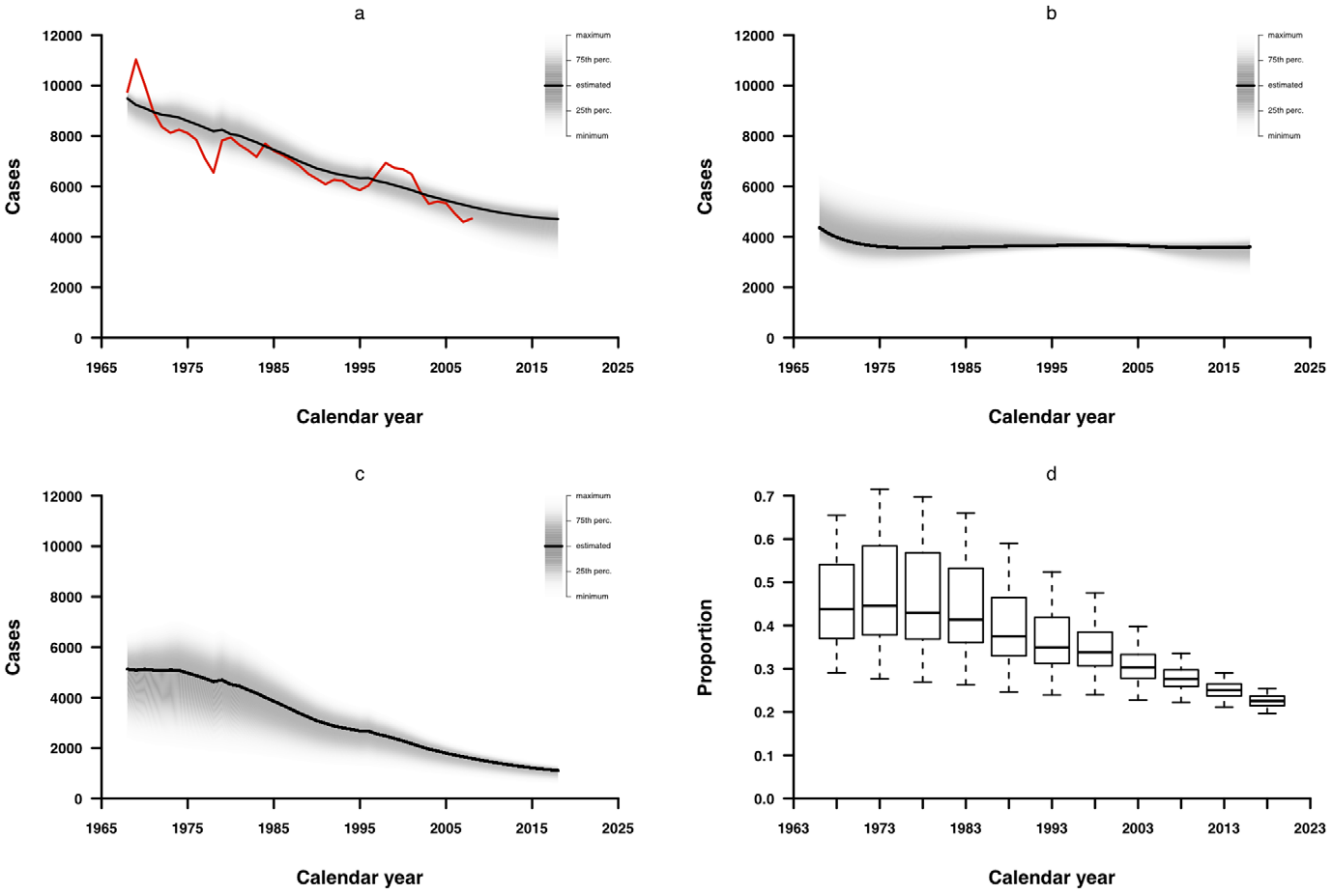
Multivariate sensitivity analysis. (a) observed TB notifications, (b) estimated reactivated TB notifications, and (c) estimated recently transmitted TB cases, each compared to fitted and predicted totals, with gray shaded areas indicating the estimated notifications under the 100 sets of fixed parameters under Latin hypercube resampling; (d) proportion of new notifications due to recent transmission at 5 year intervals from 1968 through 2018.

In one-way sensitivity analyses, none of the parameters had a substantial influence on the predicted number of TB notifications when varied within plausible ranges (data not shown). In the multivariate sensitivity analysis we found that the main inferences remain unchanged, while the proportion of cases due to recent transmission in 1968 and 2008 ranged from 29% to 65% and 22% to 34%, respectively (Figure 4). Models based on alternative relative transmission risk matrices could not fit the notified TB cases better, as shown by much higher values of AIC, however, the proportion of cases due to recent transmission did not change substantially compared with the previous model, decreasing from around 40% to 20% in 1968–2008.

## Discussion

The relative importance of recent exogenous infection/reinfection and endogenous reactivation on TB epidemics has been a controversial issue [21]–[23]. While at diagnosis it may be difficult to determine whether an individual case has resulted from recent exogenous infection/reinfection or endogenous reactivation of a long-term latent infection, the distinction is important for TB control. In this study we examined TB transmission dynamics in Hong Kong using a mathematical model [13] extended to incorporate age-structure which allowed us to attribute new cases to either recent transmission or endogenous reactivation. We found that the dominant force responsible for the attenuating declines in local TB incidence is a substantial proportion of notifications arising from endogenous reactivation of latent infections, even with a low estimated progression rate to active TB disease [24], [25]. A previous study of TB transmission dynamics in Hong Kong used a model fitted to notifications from 1967 through 1978 where the risk of infection was independent of the number of infectious cases and independent of age [25]. In that study, a scenario with a relatively high risk of disease and relatively low transmission was most similar to observed trends in notifications, suggesting that local trends are being driven by endogenous reactivation, and estimated a high risk of disease. In our model we allowed transmission to vary dynamically with the number of infectious cases, and we estimated the progression rate to active TB disease to be relatively low, with a lifetime risk of 1%–2% which is lower than the reported 5%–10% for individuals infected in the pre-chemotherapy era [26] perhaps due to general health improvements at the societal level since the 1970s or suggestive of different risks of disease progression in different settings.

Under our model, the proportion of TB cases attributable to recent transmission has substantially decreased since the 1970s. This decline may be attributed to the improvement of TB control measures in Hong Kong since the 1960s, in particular the implementation of directly observed treatment short-course (DOTS) in the late 1970s [5]. The reduction of infectious TB cases via effective treatment appears to have had a substantial impact on transmission [27]. Endogenous reactivation, however, develops from possibly long-term latent infection, and may be greatly influenced by longer-term demographic and socio-economic changes [6]. Hong Kong has been largely an immigrant community. The majority of older local residents were born and grew up in southern China, where risks of TB may have been quite different [28]. Endogenous reactivation of latent disease is also likely to be affected by rates of comorbidities including diabetes [29]. behavioural factors such as smoking [30], as well as immunosenescence [31]. Hong Kong has a long life expectancy and an aging population, many of whom may have latent infection, and therefore the burden of TB from endogenous reactivation may not be substantially reduced by the current focus on effective treatment of active TB cases with DOTS as a primary control measure.

The transmission of TB primarily depends on the infectiousness of active TB cases, the susceptibility of individuals, and contact patterns between susceptible individuals and infectious TB patients. Given the potential for different contact patterns within and between different groups, we divided the Hong Kong population into children, younger adults and older adults in our model. The estimated parameters from the model suggest that the transmission of TB is highest within younger adults and is lowest within children in Hong Kong. The estimated force of transmission in our model reflects not only contact patterns but also the transmissibility of TB within different age groups (Text S1). Although effective contacts within children in Hong Kong may not be less frequent than other age groups, and may even be more frequent, we estimated that the force of transmission of TB within children is still lower than other age groups. One possible explanation of this is that incident disease in children may be more likely to be primary disease which is less infectious than post-primary TB often observed in adults [32]. In the period studied in our model, more than 99% of children in Hong Kong received BCG vaccination at birth [5], [33], which prevents the haematogenous dissemination of tubercle bacilli in infected children [34]. Therefore another possible explanation is that BCG vaccine has led to reductions in transmission in children.

There are some limitations in our analyses. First, we assumed that the progression rates to develop active TB were constant throughout the whole study period, which might underestimate the progression rates in the earlier years and overestimate it more recently, due to socio-economic improvements or other factors. There are few data in the literature on progression rates, and without such information it is difficult to include additional parameters in our model, although we have specifically allowed for the risk of progression to be higher in the first five years after infection. Nevertheless assuming constant rates in our model was sufficient to describe TB dynamics in Hong Kong in the past four decades; in further work we may examine the support for more complicated assumptions such as period-specific progression rates. Second, we did not include partial immunity into the model, which might have led us to underestimate the role of endogenous reactivation on the transmission of TB in Hong Kong. If reinfection is less common due to partial immunity, a greater proportion of cases in older individuals would be associated with endogenous reactivation and the estimates from our model on the role of reactivation may be slightly conservative. However with low incidence rates, reinfection was relatively rare and if we had included partial immunity it is unlikely that the conclusions of our study regarding the relative importance of endogenous reactivation would have changed substantially. Third, although in Hong Kong around 65% of cases are in males [11], we neglected potentially different transmission dynamics between males and females because in our model we aimed to understand the most important elements affecting the population dynamics of TB. There are few data to explain the differences between males and females, particularly in the potentially different contact patterns historically, while there is some evidence that differential smoking rates or access to healthcare may be partially responsible [30], [35], [36]. Fourth, we allowed for age-specific mixing in our model, however if TB dynamics in Hong Kong are more complex we may have underestimated the role of re-infection in some groups [37]. The pattern of age-specific TB incidence in adults was bimodal in the 1970s and 1980s but incidence became more similar across ages after the mid-1990s. We tried to use a parsimonious model to explain the general trends in age-specific TB incidence, but we may not have fully incorporated all sources of time varying factors to capture more subtle changes in incidence. Hence the predicted fluctuations in the age-specific incidence rate under our model should not be interpreted as excess incidence at certain ages. Lastly, we fitted our model by comparing predicted cases to observed notifications, essentially assuming a 100% detection rate, which might underestimate the actual burden of TB in Hong Kong. Nevertheless, this is not likely to lead to substantial bias since Hong Kong has had a well-established TB control system for decades, and in a detailed audit the detection rate was estimated to be around 95% [38].

In conclusion, endogenous reactivation of long-term latent infection is an important component in the intermediate burden of TB in Hong Kong, currently accounting for more than 80% of new notifications. In line with the Millennium Development Goals proposed by the United Nations, the Stop TB Partnership launched the Global Plan to Stop TB in 2006 aiming to decrease TB morbidity and mortality by 50% by 2015 compared with the levels in 1990, and to eliminate TB worldwide by 2050 [39], [40]. The primary proposed means to achieve the 2015 target is an expansion of DOTS and construction and improvement of healthcare infrastructure. While a well-implemented effective treatment programme and active disease detection system may be effective in settings with high transmission, in the long-term and in developed settings it may also be important to focus on early detection of potentially reactivated disease, and screening to identify individuals with latent TB infection followed by treatment to prevent endogenous reactivation to active disease. Hong Kong has experienced rapid development since 1945, and our findings have important implications for the burden of TB in developing regions with higher levels of transmission currently. While reductions in transmission with socio-economic development and public health interventions will lead to declines in TB incidence in these regions, a high prevalence of latent infections may hinder substantial declines in burden in the longer term.

## Supporting Information

Table S1 Summary of the variables and notation used in the model.
(0.09 MB DOC)

Table S2 Correlation matrix of the estimated parameters based on data from 2003–2018.
(0.03 MB DOC)

Figure S1 The relative risk of disease progression for TB-infected individuals in different age groups.
(0.23 MB TIF)

Figure S2 The age-specific prevalence of latent tuberculosis infection in the initial state in 1961. L1, L2, L3, L4 and L5 represent the prevalence of individuals who have been infected with TB for over 1, 2, 3, 4, 5 years but had not developed active TB disease in Hong Kong prior to 1961.
(0.31 MB TIF)

Text S1 Technical details of the model.
(0.10 MB PDF)
