# Supplementary material for: The Transmission Dynamics of Tuberculosis in a Recently Developed Chinese City
Source: PLoS One. 2010 May 3;5(5):e10468. doi: 10.1371/journal.pone.0010468 (PMC2862741; doi:10.1371/journal.pone.0010468)
Supplement: Table S1 — Summary of the variables and notation used in the model. (0.09 MB DOC) [file pone.0010468.s001.doc]

Table S1. Summary of the variables and notation used in the model.

| Variable | Biological Interpretation |
| --- | --- |
| $S(a,t)$ | Number of population who have never been infected with TB with age $a$at time $t$ |
| $N(a,t)$ | Number of population with the age $a$at time $t$ |
| $N(t)$ | Total population size at time *t* |
| $L_{k}(a,t)$ | Number of population who have been infected with TB without presenting any clinical symptoms and signs with age $a$at time $t$ |
| $T_{I}(a,t)$ | Number of population who are having infectious TB disease with age $a$at time $t$ |
| $T_{N}(a,t)$ | Number of population who are having non-infectious TB disease with age $a$at time $t$ |
| $R(a,t)$ | Number of population who have been recovered from active TB disease episode with age $a$at time $t$ |
| $M_{S}(a,t)$ | Number of immigrants who have never been infected with TB with age $a$at time $t$ |
| $M_{L}(a,t)$ | Number of immigrants who have been infected with TB without presenting any clinical symptoms and signs with age $a$at time $t$ |
| $\pi(t)$ | Annual birth rate at time $t$ |
| $\lambda(a,t)$ | Force of infection for people susceptible to TB with the age of $a$at time $t$ |
| $b(a,a')$ | Transmission rate within the groups of individuals older than 35 years |
| $\sigma(t)$ | Period-specific birth rate at time *t* |
| $\alpha$ | Proportion of new infections directly developing infectious or non-infectious TB from susceptible or recovered |
| $\theta$ | Proportion of developing infectious TB directly from the susceptible or recovered |
| $\gamma$ | Proportion of developing infectious TB from latent TB infection |
| $\upsilon_{r}(i,t)$ | Annual risk of disease progression within the first 5 years after infection for individuals at age group *i* at time *t* |
| $\upsilon_{l}(i,t)$ | Annual risk of disease progression after infection for more than 5 years for individuals at age group *i* at time *t* |
| *p_r_* | Disease progress rate within the first 5 years after infection in young  adults (aged 24-40 years) |
| *p_l_* | Disease progress rate after being infected for more than 5 years in young adults (aged 24-40 years) |
| $\kappa_{i}$ | Relative risk of disease progression at age group *i*  (*i*=1 (0-8 years), 2 (9-23 years), 3 (24-40 years), 4 (41-80 years)) |
| $\omega(t)$ | Probability of relapse for recovered patients at time *t* |
| $\varphi(t)$ | Recovery rate for infectious or non-infectious TB patients at time $t$ |
| $\mu(a,t)$ | Death rate for people with the age of $a$at time $t$ |
| $\pi_{L0}$ | The prevalence of latent TB in 1961 |
| $\pi_{T0}$ | The ratio of TB prevalence to incidence cases in 1961 |
