# Supplementary material for: The Transmission Dynamics of Tuberculosis in a Recently Developed Chinese City
Source: PLoS One. 2010 May 3;5(5):e10468. doi: 10.1371/journal.pone.0010468 (PMC2862741; doi:10.1371/journal.pone.0010468)
Supplement: Table S2 — Correlation matrix of the estimated parameters based on data from 2003–2018. (0.03 MB DOC) [file pone.0010468.s002.doc]

Table S2. Correlation matrix of the estimated parameters based on data from 2003 – 2018.

|  | *b* | *M*_2_ | *M*_1_ | *α* | *p*_1_ | *p*_2_ |
| --- | --- | --- | --- | --- | --- | --- |
| *b* | 1.000 |  |  |  |  |  |
| *M*_2_ | -0.851 | 1.000 |  |  |  |  |
| *M*_1_ | -0.833 | 0.996 | 1.000 |  |  |  |
| *α* | 0.008 | -0.532 | -0.553 | 1.000 |  |  |
| *p_r_* | -0.354 | 0.788 | 0.791 | -0.931 | 1.000 |  |
| *p_l_* | 0.727 | -0.289 | -0.239 | -0.615 | 0.285 | 1.000 |
