# Supplementary figures and images for: The Transmission Dynamics of Tuberculosis in a Recently Developed Chinese City
Source: PLoS One. 2010 May 3;5(5):e10468. doi: 10.1371/journal.pone.0010468 (PMC2862741; doi:10.1371/journal.pone.0010468)

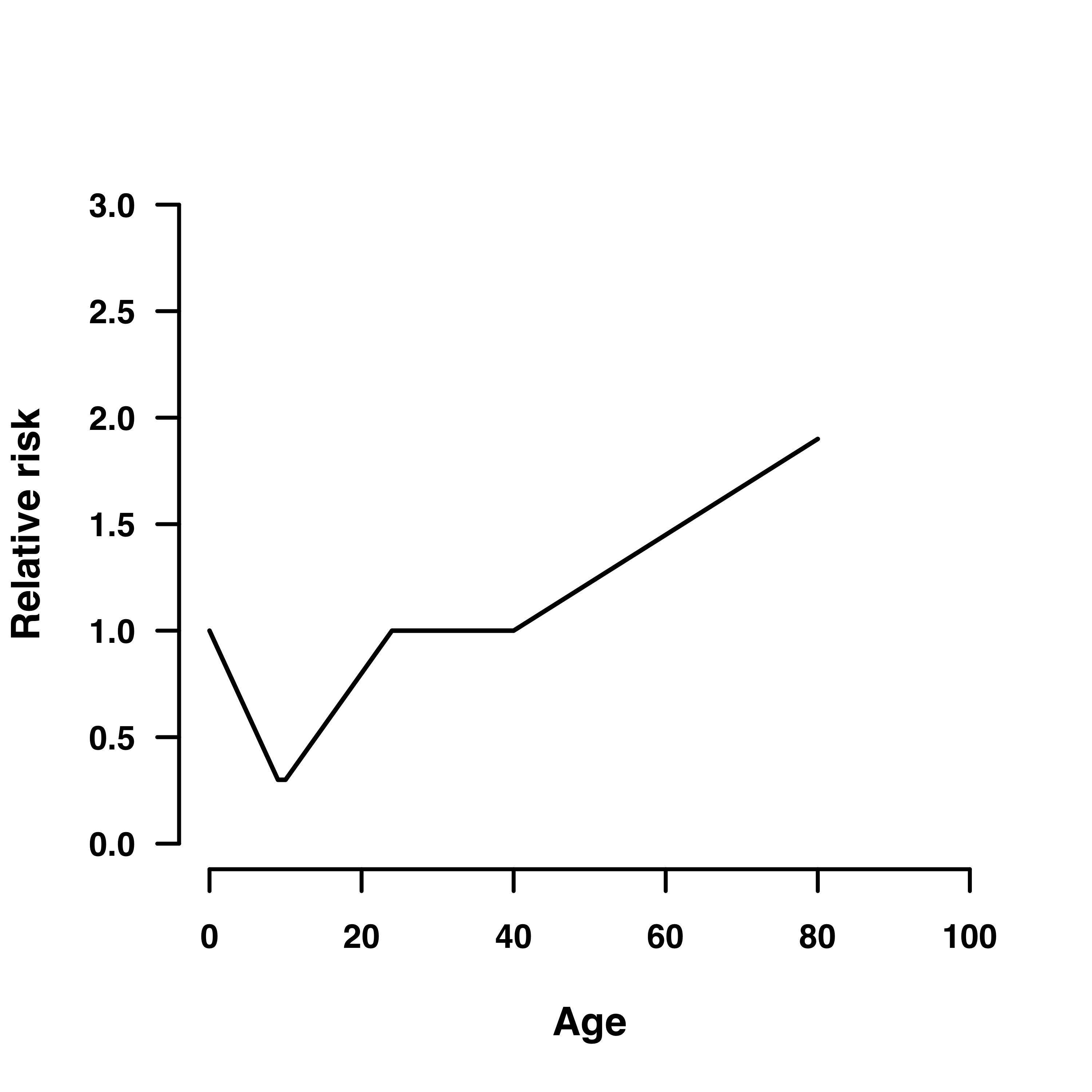

Supplement: Figure S1 — The relative risk of disease progression for TB-infected individuals in different age groups. (0.23 MB TIF) [file pone.0010468.s003.tif]

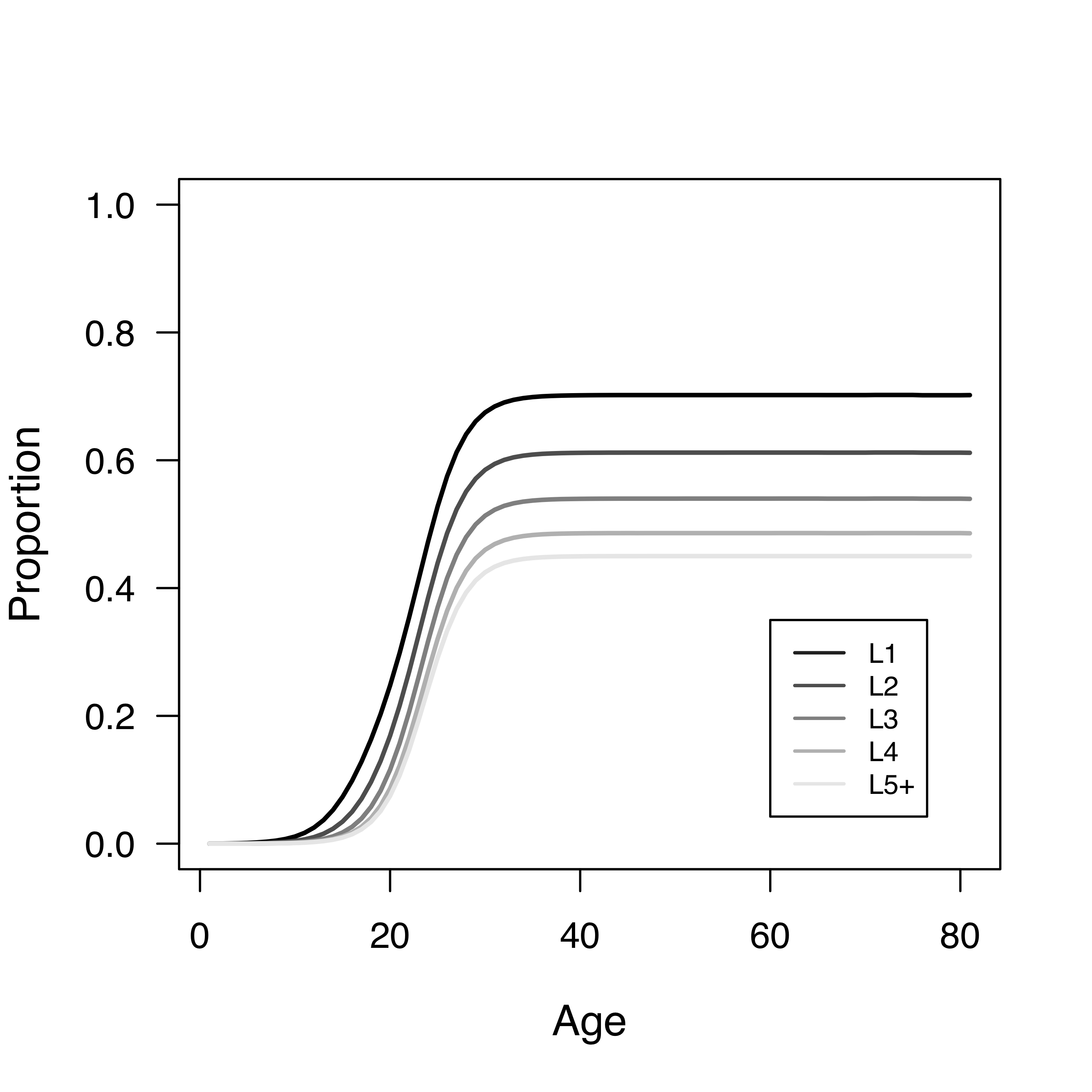

Supplement: Figure S2 — The age-specific prevalence of latent tuberculosis infection in the initial state in 1961. L1, L2, L3, L4 and L5 represent the prevalence of individuals who have been infected with TB for over 1, 2, 3, 4, 5 years but had not developed active TB disease in Hong Kong prior to 1961. (0.31 MB TIF) [file pone.0010468.s004.tif]
