# Supplementary material for: The Transmission Dynamics of Tuberculosis in a Recently Developed Chinese City
Source: PLoS One. 2010 May 3;5(5):e10468. doi: 10.1371/journal.pone.0010468 (PMC2862741; doi:10.1371/journal.pone.0010468)
Supplement: Text S1 — Technical details of the model. (0.10 MB PDF) [file pone.0010468.s005.pdf]

Supplementary Information

We constructed a population based deterministic model with age-structured compartments to describe tuberculosis (TB) transmission dynamics in Hong Kong. We formulated the model structure to fully incorporate the major determinants of TB transmission and dynamics, such as demographic distribution or changes due to immigration or emigration, and the pathogenesis of tuberculosis featured by a possible long latency, progressing into active infectious state through different mechanisms of reactivation and reinfection. We used eight active compartments (Figure 1) to represent the states involved in the TB transmission dynamics and one compartment to represent the absorbing state of death. We provided PDE equations (Appendix page 4-6) to describe transition dynamics of the model, and approximated the PDE by finite difference methods in the view of the complexity of the model and limited resolution of available data. All the fixed parameters and estimated parameters are summarized in Table S1.

Individuals are born without infection and are assumed susceptible (S) to infection. Assuming infectiousness is constant throughout the infectious period, the force of infection $\lambda(a,t)$ at time t for individuals susceptible to TB with age a is proportional to: an infection parameter, b; age-specific relative transmission parameters, $M_{i,j}$; and the proportion of infectious individuals of aged a at time t, $T_I(a,t)/N(t)$ (i.e, frequency-dependent transmission). The force of infection is:

$$\lambda(a,t) = b\int_{k=0}^{a_{max}} M_{i(k),j(a)} T_I(k,t)/N(t)\mathrm{d}k, \text{ where}$$

$$i(x), j(x) = \begin{cases} 1 & \text{when } 0 \leq x < 16 \\ 2 & \text{when } 16 \leq x < 31 \\ 3 & \text{when } 31 \leq x \leq 80 \end{cases}$$

a_{max} is the maximal age of interest in the model and $M_{i,j}$ (dependency on a suppressed for simplicity) is given by the following matrix which is referred to the study conducted by Del Valle SY et al*.

The derived matrix is:

Infectious	Susceptible		
	0-15y	16-30y	> 30y
0-15y	$M_{1,1}$	$M_{1,2}$	$M_{1,3}$
16-30y	$M_{2,1}$	$M_{2,2}$	$M_{2,3}$
> 30y	$M_{3,1}$	$M_{3,2}$	$M_{3,3}$

$$M_1 = M_{1,1} = M_{2,1} = M_{3,1} = M_{1,2} = M_{1,3}$$
$$M_2 = M_{2,2}$$
$$M_3 = M_{2,3} = M_{3,2} = M_{3,3}$$

After infection, an individual may become an active TB case directly with a probability α. There is a probability of θ that the active TB case will be a recent infected TB case (RIITB) and a probability $1-\theta$ of being a recent infected non-infectious TB case (RINTB). Otherwise, with a probability $1-\alpha$ the individual develops latent infection, and may later develop active infectious TB (RIITB) or non-infectious TB (RINTB) at a rate of $\gamma\nu_r(a)$ and $(1-\gamma)\nu_r(a)$ respectively for individuals with age a, or may continue to have latent infection without presenting any clinical symptoms or signs of active disease. In such case, an individual of age a is described by the model as having recent latent TB infection (RLTBI) and will develop active TB with probability $\kappa(a)p_r$. RLTBI is a transient state and after 5 years of infection the individual transits to the long-term latent TB infection (LLTBI) state where it is possible to develop active TB (reactivated infectious TB, RAITB or reactivated non-infectious TB, RANTB) with probability $\kappa(a)p_l$ over the remainder of their lifetime, or to be reinfected to directly develop active TB with probability $\alpha\theta\lambda(a,t)$ (to RIITB) and probability $\alpha(1-\theta)\lambda(a,t)$ (to RINTB), or go back to the state of RLTBI with probability $(1-\alpha)\lambda(a,t)$. The relations between $\nu_r(a), \nu_l(a), p_r$ and p_l are given by:

$$\nu_r(a) = \kappa(a)p_r \quad (0 \leq a \leq 80)$$
$$\nu_l(a) = \kappa(a)p_l \quad (0 \leq a \leq 80)$$

The recovery rate for active TB cases at time t is given by $\phi(t)$. A recovered individual does not have immunity and can be reinfected and directly develop active TB disease, becoming recent reinfected infectious TB, RIITB or non-infectious TB, RINTB, or stay at the latent state for a while, developing active TB disease later. In both cases, the infection rate for recovered individuals is assumed to be the same as that of susceptible individuals. A recovered individual may also relapse to become a reactivated infectious TB case (RAITB) or reactivated non-infectious TB case (RANTB), both at a rate of ω.

1. The following equations denote the transition rates under our model.

$$(1.1)\qquad \frac{\partial S}{\partial t}(0,t) = \overbrace{\delta(t)N(t)}^{Newborn} - \overbrace{(1-\alpha)\lambda(0,t)S(a,t)}^{S\to RLTBI_1} - \overbrace{\alpha\theta\lambda(0,t)S(0,t)}^{S\to RIITB} - \overbrace{\alpha(1-\theta)\lambda(0,t)S(0,t)}^{S\to RINTB} - \overbrace{\mu(0,t)\left(S(0,t)+m_S(0,t)\right)}^{Deaths}$$

$$(1.2)\qquad \frac{\partial S}{\partial t}(a,t) + \frac{\partial S}{\partial a}(a,t) = -\overbrace{(1-\alpha)\lambda(a,t)(S(a,t)+m_S(a,t))}^{S\to RLTBI_1} - \overbrace{\alpha\theta\lambda(a,t)(S(a,t)+m_S(a,t))}^{S\to RIITB} - \overbrace{\alpha(1-\theta)\lambda(a,t)(S(a,t)+m_S(a,t))}^{S\to RINTB}$$
$$+ \overbrace{m_S(a,t)}^{Susceptible\ migrants} - \overbrace{\mu(a,t)\left(S(a,t)+m_S(a,t)\right)}^{Deaths}, \quad (a>0)$$

$$(2)\qquad \frac{\partial L_1}{\partial t}(a,t) + \frac{\partial L_1}{\partial a}(a,t) = \overbrace{(1-\alpha)\lambda(a,t)(S(a,t)+m_S(a,t))}^{S\to RLTBI_1} + \overbrace{(1-\alpha)\lambda(a,t)R(a,t)}^{R\to RLTBI_1} - \overbrace{\gamma\nu_r(a)L_1(a,t)}^{RLTBI_1\to RIITB} - \overbrace{(1-\gamma)\nu_r(a)L_1(a,t)}^{RLTBI_1\to RINTB}$$
$$- \overbrace{L_1(a,t)(1-\nu_r(a)-\mu(a,t))}^{RLTBI_1\to RLTBI_2} + \overbrace{(1-\alpha)\lambda(a,t)(L_5(a,t)+m_L(a,t))}^{LLTBI\to RLTBI_1} - \overbrace{\mu(a,t)L_1(a,t)}^{Deaths}$$

$$(3)\qquad \frac{\partial L_k}{\partial t}(a,t) + \frac{\partial L_k}{\partial a}(a,t) = \overbrace{L_{k-1}((a-1),(t-1))(1-\nu_r(a-1)-\mu((a-1),(t-1)))}^{RLTBI_{k-1}\to RLTBI_k} - \overbrace{L_k(a,t)(1-\nu_r(a)-\mu(a,t))}^{RLTBI_k\to RLTBI_{k+1}} - \overbrace{\gamma\nu_r(a)L_k(a,t)}^{RLTBI_k\to RIITB}$$
$$- \overbrace{(1-\gamma)\nu_r(a)L_k(a,t)}^{RLTBI_k\to RINTB} - \overbrace{\mu(a,t)L_k(a,t)}^{Deaths} \qquad (k=2,3,4)$$

$$(4)\quad \frac{\partial L_5}{\partial t}(a,t)+\frac{\partial L_5}{\partial a}(a,t) = \overbrace{L_4((a-1),(t-1))(1-\nu_r(a-1)-\mu((a-1),(t-1)))}^{RLTBI_4\rightarrow LLTBI} - \overbrace{(\alpha\theta\lambda(a,t)+\gamma\nu_l(a))(L_5(a,t)+m_L(a,t))}^{LLTBI\rightarrow RIITB/RAITB}$$
$$-\overbrace{(\alpha(1-\theta)\lambda(a,t)+(1-\gamma)\nu_l(a))(L_5(a,t)+m_L(a,t))}^{LLTBI\rightarrow RINTB/RANTB}$$
$$-\overbrace{(1-\alpha)\lambda(a,t)(L_5(a,t)+m_L(a,t))}^{LLTBI\rightarrow RLTBI_1}+\overbrace{m_L(a,t)}^{Migrated\ LLTBI}-\overbrace{\mu(a,t)(L_5(a,t)+m_L(a,t))}^{Deaths}$$

$$(5)\quad \frac{\partial T_I}{\partial t}(a,t)+\frac{\partial T_I}{\partial a}(a,t) = \overbrace{\alpha\theta\lambda(a,t)(S(a,t)+m_S(a,t))}^{S\rightarrow RIITB}+\overbrace{\sum_{k=1}^{4}\gamma\nu_r(a)L_k(a,t)}^{RLTBI_k\rightarrow RIITB}+\overbrace{(\alpha\theta\lambda(a,t)+\gamma\nu_l(a))(L_5(a,t)+m_L(a,t))}^{LLTBI\rightarrow RIITB/RAITB}$$
$$+\overbrace{(\alpha\theta\lambda(a,t)+\omega)R(a,t)}^{R\rightarrow RIITB/RAITB}-\overbrace{\phi(t)T_I(a,t)}^{RIITB/RAITB\rightarrow R}-\overbrace{\mu(a,t)T_I(a,t)}^{Deaths}$$

$$(6)\quad \frac{\partial T_N}{\partial t}(a,t)+\frac{\partial T_N}{\partial a}(a,t) = \overbrace{\alpha(1-\theta)\lambda(a,t)(S(a,t)+m_S(a,t))}^{S\rightarrow RINTB}+\overbrace{\sum_{k=1}^{4}(1-\gamma)\nu_r(a)L_k(a,t)}^{RLTBI_k\rightarrow RINTB}+\overbrace{(\alpha(1-\theta)\lambda(a,t)+(1-\gamma)\nu_l(a))L_5(a,t)}^{LLTBI\rightarrow RINTB/RANTB}$$
$$+\overbrace{(\alpha(1-\theta)\lambda(a,t)+\omega)R(a,t)}^{R\rightarrow RINTB/RANTB}-\overbrace{\phi(t)T_N(a,t)}^{RINTB/RANTB\rightarrow R}-\overbrace{\mu(a,t)T_N(a,t)}^{Deaths}$$

$$(7)\quad \frac{\partial R}{\partial t}(a,t)+\frac{\partial R}{\partial a}(a,t) = \overbrace{\phi(t)T_I(a,t)}^{RIITB/RAITB\rightarrow R}+\overbrace{\phi(t)T_N(a,t)}^{RINTB/RANTB\rightarrow R}-\overbrace{(1-\alpha)\lambda(a,t)R(a,t)}^{R\rightarrow RLTBI_1}-\overbrace{(\alpha\theta\lambda(a,t)+\omega)R(a,t)}^{R\rightarrow RIITB/RAITB}$$
$$-\overbrace{(\alpha(1-\theta)\lambda(a,t)+\omega)R(a,t)}^{R\rightarrow RINTB/RANTB}-\overbrace{\mu(a,t)R(a,t)}^{Deaths}$$

2. The numbers of cases due to recent transmission and reactivation

(1) Recent transmission

$$\overbrace{\alpha\theta\lambda(a,t)(S(a,t)+m_S(a,t))}^{S\to RIITB}+\overbrace{\alpha(1-\theta)\lambda(a,t)(S(a,t)+m_S(a,t))}^{S\to RINTB}+\overbrace{\sum_{k=1}^{4}\gamma\nu_r(a)L_k(a,t)+\alpha\theta\lambda(a,t)(L_5(a,t)+m_L(a,t))}^{RLTBI_k/LLTBI\to RIITB}$$
$$+\overbrace{\sum_{k=1}^{4}(1-\gamma)\nu_r(a)L_k(a,t)+\alpha(1-\theta)\lambda(a,t)(L_5(a,t)+m_L(a,t))}^{RLTBI_k/LLTBI\to RINTB}+\overbrace{\alpha\theta\lambda(a,t)R(a,t)}^{R\to RIITB}+\overbrace{\alpha(1-\theta)\lambda(a,t)R(a,t)}^{R\to RINTB}$$

(2) Reactivation

$$\overbrace{\gamma\nu_l(a)(L_5(a,t)+m_L(a,t))}^{LLTBI\to RAITB}+\overbrace{(1-\gamma)\nu_l(a)(L_5(a,t)+m_L(a,t))}^{LLTBI\to RANTB}+\overbrace{\omega R(a,t)}^{R\to RAITB}+\overbrace{\omega R(a,t)}^{R\to RANTB}$$

Initial state

For the initial states of the model in year 1961, we assumed that the prevalence of latent TB infection in different age groups followed a logistic distribution with the prevalence of TB being 0.7 in people aged over 35 years old. The numbers of people with active TB disease in different age groups were derived from the TB notification data in Hong Kong by assuming that the number of prevalent active TB cases is about 2.5 times the number of notified cases with the same age. The distribution of recovered individuals from active TB disease in different age groups was also assumed to follow a logistic distribution.

We assumed the prevalence of individuals who have been infected with TB for over 1, 2, 3, 4, 5 years but not developed active TB disease in Hong Kong in 1961 followed logistic distributions, $L_1(t), L_2(t), L_3(t), L_4(t), L_{5+}(t)$. The prevalence of individuals who have been infected for over 1 year but less than 2 years is $L_1(t) - L_2(t)$, over 2 years but less than 3 years is $L_2(t) - L_3(t)$, over 3 years but less than 4 years is $L_3(t) - L_4(t)$, and over 4 years but less than 5 years is $L_4(t) - L_{5+}(t)$ in 1961.

The parameters used in the logistic function are:

L_{maxi}, maximum of the logistic function ($i = 1, 2, 3, 4, 5$).

$a = 7,\ b = 0.05,\ c = 17,$ parameters for the curvature of the logistic function.

$$k = \log \frac{L_{max}}{L_{max} b - 1}$$

$m = \log(\frac{1}{1-\delta} - 1),$ where δ is a small number (we used 0.01 in our model)

$n_j =$ the age of subjects

$$L_1 = \frac{L_{max1}}{1 + \exp(k)\exp(-\frac{k-m}{c}(n_j - a))}$$

$$L_2 = \frac{L_{max2}}{1 + \exp(k)\exp(-\frac{k-m}{c}(n_j - 1 - a))}$$

$$L_3 = \frac{L_{max3}}{1 + \exp(k)\exp(-\frac{k-m}{c}(n_j - 2 - a))}$$

$$L_4 = \frac{L_{max4}}{1 + \exp(k)\exp(-\frac{k-m}{c}(n_j - 3 - a))}$$

$$L_{5+} = \frac{L_{max5}}{1 + \exp(k)\exp(-\frac{k-m}{c}(n_j - 4 - a))}$$

The age-specific mid-year populations were obtained from official statistics published

by the Census and Statistics Department of the Hong Kong government.

Population movement

Considering potential impact of migrated population on the incidence of TB in Hong Kong, we incorporated immigrants and emigrants in our model. We derived the number of migrants aged a at time t in Hong Kong in 1961-2008 from the officially published data by the Census and Statistics Department. The net movement of population was calculated with the equations below:

$$P_e(a,t) = P(a-1,t-1) - D(a-1,t-1) \quad (1)$$

$$m(a,t) = P(a,t) - P_e(a,t) \quad (2)$$

$P_e(a,t)$ is the estimated number of population aged a at time t in Hong Kong if there was no population movement at that age group and that time. $P(a-1,t-1)$ is the reported number of individuals aged $a-1$ at time $t-1$ and $D(a-1,t-1)$ is the number of individuals aged $a-1$ died at time $t-1$. $m(a,t)$ is the estimated number of individuals aged a moved at time t.

As the number of population is reported by every 5-year age group in Hong Kong, we averaged the 5-year age group data to achieve the approximate number of population in each age group. However, the way to calculate the number of age-specific population would potentially make the difference in the numbers in the $5a^{th}$ and $(5a+1)^{th}$ age groups extraordinarily higher than other adjacent age groups. To avoid these sudden leaps, we averaged the $m(a,t)$ in every 5 years of age to get the number of migrants aged a at time t. At the end, the net movement of population in each 5-year age group in our model is exactly the same as officially published data.

To simplify the model, we assumed that the migrants were either susceptible or long-term latently infected. If $m(a,t)$ in equation (2) is positive, it means there are net immigrants in the according age group; if negative, suggesting net emigrants. We also assumed the prevalence of TB in immigrants and emigrants are different as they originated from places with different disease prevalence. The prevalence of TB in the mainland China (especially Southern China) was assumed higher than that in Hong

Kong due to different public health infrastructures and also suggested by published studies.

Disease progression rate

We assumed that the disease progression rate within or more than 5 years after TB infection varies for infected individuals with different age, and the relative risk of disease progression for infected people in the reference group (aged 24) is 1. The assumptions here were based on a prospective study conducted by Chan-Yeung M et al.[†] and the reviewed results from Marais BJ et al.[♯] The variation of relative risk of disease progression with age is illustrated in Figure S2.

Parameter estimation

We estimated 6 key parameters relating to the transmission dynamics. We also estimated 1 overdispersion parameter for the distribution specifying the likelihood. Estimation was carried out using the `optim` function in R by maximizing a negative binomial based likelihood as the simulated age-specific TB notifications were fitted against the observed age-specific TB notifications. Multiple sets of initial values were used to ensure the obtained solution was optimal in the plausible solution space. The log likelihood is obtained by grouping simulated data into age groups and years, and given by:

$$\ell(p) = \sum_{t=1}^{T} \sum_{a=0}^{a_{max}} \log P_{negbin}(m(a,t); m^*(a,t|p), k)$$

where a_{max} is the maximal age considered in the model, T is the study period, $m^*(a,t|p)$ is the simulated TB notifications in age group a at time t based on paramaters p, $m(a,t)$ is the observed TB notifications in age group a at time t and k is the dispersion parameter of the negative binomial distribution. P_{negbin} is the probability mass function of a negative binomial distribution. In the model, we set $T = 58$ and $a_{max} = 80$.

To evaluate the uncertainty in the parameter estimates, we constructed marginal 95% confidence intervals. We used the function `hessian` in R to evaluate numerically the information matrix based on the likelihood function. The variance covariance

matrix of the estimated parameters was then derived and we identified the confidence hyperellipsoid p which satisfied the following relation:

$$(p - p^*)V_p^{-1}(p - p^*) = \chi_7^2(\alpha)$$

where p^* is the estimated parameter vector, V_p is the variance-covariance matrix, χ_7^2 is the chi-squared distribution with 7 degrees of freedom and α is the significance level. For ease of presentation we took the boundary of the hyperellipsoid with respect to each estimated parameter as the marginal confidence intervals.

The dispersion parameter was estimated to be 15.3 (95% CI: 13.8-17.2). Other parameter estimates are summarized in Table 2.

Sensitivity analysis

We performed one-way sensitivity analysis to examine the influence of each of the fixed parameters on the trends in TB notifications predicted by the model. We varied the proportion of active TB disease which is infectious (θ), the proportion of active TB disease from latent TB infection (RLTBI or LLTBI) which is infectious (γ), the probability of relapse for recovered patients (ω), the recovery rate for TB patients in 1961 (ϕ_0), the prevalence of latent TB in 1961 (P_{L0}), and the ratio of TB prevalence to incidence in 1961 (π_{T0}). Each parameter was varied between minimum and maximum plausible values as determined from local data or the literature (Table 1).

Further, to assess the combined effect of the above parameters on our estimates, a multivariate sensitivity analysis based on Latin hypercube sampling was carried out. We subdivided the range for each variable as specified in Table 1 into equiprobable intervals, based on a uniform distribution. We then simulated 100 sets of samples in which each variable was drawn randomly and without replacement from these intervals. Based on the samples we simulated the number of annually produced active TB cases, cases due to recent transmission and endogenous reactivation and the proportion of cases from recent transmission in each year as shown in Figure 4.

Model validation

Table S2 shows the correlation matrix of the estimated parameters, from which we observed high dependency between transmission related variables b, M_1, and M_2. The negative correlation between α and p_r, p_l indicates that the model was able to maintain the tradeoff between the risk of directly developing active TB and disease progression rate from latent infection to generate trends in TB cases consistent with the data. All of the correlations are consistent with the working TB transmission dynamics conditioned on the given data.

Supplementary references

* Del Valle SY, Hyman JM, Hethcote HW, et al. Mixing patterns between age groups in social networks. *Social Networks* 2007; 29(4): 539-554.

† Chan-Yeung M, Kam KM, Leung CC, et al. Population-based prospective molecular and conventional epidemiological study of tuberculosis in Hong Kong. *Respirology* 2006; 11(4): 442-448.

♯ Marais BJ, Gie RP, Schaaf HS, et al. The clinical epidemiology of childhood pulmonary tuberculosis: a critical review of literature from the pre-chemotherapy era. *Int J Tuberc Lung Dis* 2004; 8(3): 278-285.
